# A decade of China’s health silk road: policy review for global health governance and SDG partnerships

**DOI:** 10.3389/fpubh.2025.1676960

**Published:** 2025-09-04

**Authors:** Chao Wang, Shimin Huang, Nicholas Lassi, Xiaohan Zhang

**Affiliations:** ^1^Institute for Global Health and Regulatory Science, Zhejiang Chinese Medical University, Hangzhou, China; ^2^Guanghua Law School, Zhejiang University, Hangzhou, China; ^3^School of Law, Southwest University of Political Science and Law, Chongqing, China; ^4^School of Law, Hangzhou City University, Hangzhou, China

**Keywords:** China, health silk road, health diplomacy, right to health, sustainable development, global health governance

## Abstract

The tenth anniversary of China’s Health Silk Road (HSR) offers a timely opportunity to review its contributions and challenges in advancing global health governance and international public health cooperation. As important health-related global public goods (GPGs), the HSR has sought to promote equitable access to health resources, reduce disparities among partner countries, and strengthen international collaboration in line with the UN Sustainable Development Goals (SDGs). This policy and practice review critically analyzes the governance structure, policy mechanisms, and implementation experiences of the HSR, using policy documents, international organization reports, and comparative case analysis. The review identifies central challenges, including regulatory fragmentation, inconsistent recognition of medical qualifications, and varying standards for health practices across diverse cultural and political contexts. It emphsizes the need for more transparent, inclusive, and rule-based governance frameworks that generates mutual trust and integration of non-state actors. The paper offers policy recommendations to strengthen cross-border cooperation, promote mutual learning, and deepen international partnerships, aiming to inform the future development of more transparent, inclusive, and rule-based global health governance rooted in diverse regional experiences.

## 1 Introduction

The Health Silk Road (HSR), now marking its tenth anniversary, has become a significant component of China’s efforts to strengthen global health governance and further international public health partnerships. Launched in 2016 as an extension of the Belt and Road Initiative (BRI), the HSR aims to promote more equitable access to health resources, advance capacity building, and reduce disparities among participating countries, particularly those in the Global South and the Association of Southeast Asian Nations (ASEAN), where health system diversity and persistent gaps make these countries both key beneficiaries and important testing grounds for HSR cooperation. Its development reflects the growing importance of non-Western and cross-regional approaches in international health policy.

The right to health is enshrined in international treaties and the Constitution of the World Health Organization (WHO), affirming that every individual is entitled to the highest attainable standard of health without discrimination (1, 2). However, the COVID-19 pandemic has exposed persistent vulnerabilities in both national health systems and global health governance, underscoring the urgent need for more effective and inclusive international cooperation (3). These challenges are especially acute in low- and middle-income countries, where gaps in infrastructure, resources, and policy coordination often hinder timely and equitable health responses.

Against this backdrop, over the past decade the HSR has produced a series of cooperation agreements, capacity-building programs, and emergency medical aid projects across Asia, Africa, and other regions (4, 5). Initiatives have included supporting hospital construction, establishing disease control centers, and providing critical medical supplies during public health crises (6, 7). The HSR has also promoted the exchange of knowledge and experience, contributing to the development of public health systems in partner countries. Despite these achievements, the HSR faces increasingly notable governance and policy challenges that limit its effectiveness and broader impact. These include the lack of a systematic global health strategy (8), fragmented regulatory frameworks, inconsistent standards for medical practice and qualification recognition, and varying healthcare philosophies among partner countries (9, 10). In addition, skepticism regarding the transparency and motivations of China’s health cooperation remains a barrier to deeper and more trusted partnerships. In fact, outbreaks of other infectious diseases such as Ebola, Nipah, and Zika have repeatedly demonstrated the necessity of robust, rule-based international health frameworks capable of coordinated action and rapid response (11, 12). employing international legal norms (including health, human rights, and environmental standards) will be critical to improving the HSR’s capacity to address complex and evolving global health risks (13).

In the academic literature, a limited but increasing number of scholars have examined the HSR as an emerging component of global health governance, emphasizing its role in supporting health systems, facilitating international health collaboration, and responding to the COVID-19 pandemic (14). A latest analysis by Santiago and Duarte highlights the evolving role of the HSR in China’s approach to governance and multilateralism, arguing that the initiative represents a strategic adjustment toward enhancing China’s profile as a global health player (15). Nevertheless, critical perspectives have raised concerns about transparency, regulatory harmonization, and long-term sustainability. The WHO remains the central actor in global health governance, shaping international norms and coordinating responses to transnational health challenges. Recent studies have also noted the growing competition between China and the US in this arena, with each advancing distinct models of global health engagement and influence (16). The shifting environment of global health governance (exemplified by the US withdrawal from the WHO) has shown the complexity of integrating the HSR within the existing multilateral health system (17). Despite these advances, rigorous policy reviews of the HSR’s decade-long trajectory and its implications for global health governance remain limited.

As the HSR enters its second decade, there is a pressing need to reflect on its governance structure, implementation experiences, and policy lessons. Since this ambitious initiative reflects China’s emphasis on health diplomacy as a tool to strengthen its global soft power and shape international norms in health-related fields, this review aims to critically assess the strengths and challenges of the HSR from a public health policy perspective, and to provide recommendations for building more effective, transparent, and inclusive global health governance. It seeks to answer the research question: how has the HSR contributed to strengthening health systems, promoting international health cooperation, and advancing global health governance; what key challenges and opportunities have emerged over the past decade of its implementation; and how can the HSR further reform itself to advance rule-based governance in the future? By employing a health diplomacy framework and viewing the HSR as a manifestation of soft power (18, 19), we critically analyze how China uses international health cooperation to build influence, legitimacy, and partnerships within global health governance structures. The ultimate goal is to support the realization of the universal right to health and accelerate progress toward the UN’s Sustainable Development Goals, especially SDGs 3 (Good Health and Well-being), in diverse regional contexts.

## 2 Materials and methods

This policy and practice review aims to analyze the governance structure, policy mechanisms, and implementation experience of the HSR over the past decade, with a particular focus on its implications for global health governance and partnerships. The methodology combines qualitative document analysis and comparative policy review to identify both achievements and challenges in HSR practice.

Data and materials for this review were collected from a wide range of credible sources, including official policy documents from Chinese government agencies (such as the State Council, National Development and Reform Commission, Ministry of Foreign Affairs, and National Health Commission), as well as the Pharmacopoeia of the People’s Republic of China. International organization reports and guidelines were obtained from bodies such as the WHO, WTO, CDCs, African Union, and the ASEAN. Supplementary policy papers and academic research were drawn from institutions including the ASEAN-China Centre, Boston University’s Global Development Policy Center, and the Center for Strategic and International Studies (CSIS). Media coverage was referenced from outlets such as Xinhua News Agency, CGTN, and China Daily.

Materials were selected based on credibility, publication date, and relevance to the governance, policy implementation, and cross-border cooperation of the HSR. Emphasis was placed on documents published after the launch of the HSR in 2016.

A qualitative thematic analysis was conducted to identify key themes, governance challenges, and policy gaps in HSR implementation. Comparative analysis was used to assess the HSR’s alignment with international health governance frameworks and best practices, such as those of the WHO, the UNDP, the Joint UN Programme on HIV/AIDS (UNAIDS), the Gavi on vaccine, the ECDC of European countries, the Pan American Health Organization (PAHO), and their relevant international legal instruments and mechanisms. Attention was given to issues of regulatory harmonization, medical qualification recognition, and mechanisms for cross-border cooperation. Human rights standards, environmental considerations, and health equity were also evaluated as part of the HSR’s policy context. These approaches make it possible to synthesize policy lessons from the first decade of the HSR, and to provide targeted recommendations for strengthening future international health collaboration, governance, and progress toward Sustainable Development Goals.

## 3 Results

### 3.1 Global Health cooperation under the HSR framework

Since its official launch in 2016, the HSR has served as an important platform for promoting international health cooperation, particularly among countries participating in the Belt and Road Initiative (BRI) (20). Over the past decade, the HSR has been instrumental in the signing of numerous health cooperation agreements between China and countries across Asia, Africa, and other regions, as well as with international and regional organizations such as the African Union and ASEAN (21–23). These partnerships have also extended to non-state actors, including non-governmental organizations and foundations (24), reflecting a growing openness to diverse forms of health collaboration.

Key activities under the HSR framework have included the construction and upgrading of hospitals and public health infrastructure, the establishment of Centers for Disease Control and Prevention (e.g., the Africa CDC), and large-scale medical aid initiatives. Notably, China has dispatched thousands of medical professionals to African and Asian partner countries, providing direct healthcare services and capacity-building support (25). In addition, the HSR has promoted the exchange of medical knowledge and technology, including the introduction of traditional Chinese medicine (TCM) centers in several countries (26) and the establishment of joint innovation hubs (27).

During the COVID-19 pandemic, the HSR played a visible role in global emergency response efforts. China provided essential medical supplies, technical expertise, and vaccine support to many BRI countries facing urgent needs and limited access to health resources (28). These contributions were recognized by international organizations such as the WHO and Africa CDC for their role in strengthening global health security. The establishment of vaccine production centers and support for cold chain logistics in Southeast Asia further demonstrated the HSR’s potential to address critical gaps in global public health preparedness (29). In the meanwhile, the HSR’s efforts have sometimes been met with skepticism regarding the underlying motivations and long-term impact of China’s health assistance (30). Geopolitical tensions and differences in public health philosophies among partner countries have at times complicated cooperation and policy implementation.

In short, the first decade of the HSR has demonstrated both the opportunities and complexities of advancing global health cooperation through a non-Western, partnership-oriented framework. While the HSR has contributed to capacity building, infrastructure development, and emergency response in many countries, ongoing challenges around regulation, trust, and policy alignment remain central to its future evolution as a platform for international health governance and SDG3 progress.

### 3.2 Challenges in HSR governance

While the HSR offers important opportunities for international health cooperation, it has also attracted critical perspectives and faced significant challenges before being recognized as a globally accepted institution for providing GPGs for health. The international community’s perception of the HSR remains ambivalent, with some countries expressing ideological or geopolitical reservations (31, 32). In particular, similar to the economic and trade aspects of the BRI, which have faced accusations of “debt traps” and “economic colonialism” (33, 34), a portion of HSR’s health projects have been stigmatized by some national governments (35). Critics argue that HSR-related projects, particularly large-scale infrastructure and technology transfers, may increase the financial burden on recipient countries, leading to unsustainable debt levels. Such concerns are especially salient in low- and middle-income countries, where limited fiscal space may constrain long-term repayment capacity. Some observers warn that this could generate dependency on Chinese financing, technology, and expertise, potentially undermining local capacity-building and autonomy (36, 37). Addressing these challenges requires greater transparency in project financing, inclusive stakeholder engagement, and an emphasis on sustainable, context-appropriate solutions under the HSR framework, to ensure that cooperation supports genuine health system strengthening rather than dependency.

The COVID-19 pandemic also exposed governance challenges within the HSR. One major issue is the divergence in health philosophies among participating countries, stemming from differences in political systems, cultural backgrounds, and levels of economic development. These differences impede policy communication and cooperation. Many HSR countries have inadequate health infrastructure and insufficient financial investment in healthcare systems, limiting their capacity to address public health emergencies effectively (38, 39). Pandemic prevention and control measures vary significantly from country to country, reducing the effectiveness of global coordination against epidemics. For example, countries adopted divergent strategies in response to the COVID-19 outbreak. Some countries have implemented strict “containment” measures, with China enforcing a rigorous “dynamic zero-COVID-19” policy to suppress infections quickly. In contrast, other countries pursued “mitigation” strategies, opting for moderate approaches focused on reducing virus transmission rates and enabling coexistence with the virus through natural immunity. These contrasting strategies show the difficulty of facilitating coordination among HSR partner states.

### 3.3 Legal and regulatory barriers

A notable barrier to HSR effectiveness is the inconsistent medical standards and regulations among participating countries. Deviating certification and licensing standards often hinder the deployment of medical personnel and aid. Some HSR countries have more stringent physician certification processes than others (40), limiting the recognition of Chinese practitioners’ qualifications (41). For example, in Guyana, which closely follows the British medical system, foreign doctors must pass a rigorous qualification process, including an English proficiency assessment, knowledge of the Guyana Medical Practitioners Act, and evaluations of their medical specialties (42).

Traditional Chinese medicine (TCM) also faced substantial obstacles in many HSR-participating countries where health regulatory frameworks are rooted in Western medical systems. This has limited the recognition of TCM’s legal and professional status (43). For instance, while TCM has a presence in Malaysian markets, it lacks legal protections and regulatory clarity (44). During the COVID-19 pandemic, China promoted the TCM capsule Lianhua Qingwen as a treatment, but it was deemed illegal and unusable in many countries (45). As of June 2025, the International Organization for Standardization (ISO/TC249) has published only 123 TCM items (46), compared to the 5,911 standards for Chinese herbal medicines published by the Pharmacopoeia of the People’s Republic of China (47).

### 3.4 Coordination challenges

The HSR lacks a central coordinating body for global-level information sharing. Effective global health governance requires a supranational system to align policies, legal requirements, and technical standards for disease control, vaccine development, and equitable resource access. China’s approach has been primarily bilateral and project-specific, with limited regional or international organization involvement, resulting in fragmented information sharing. This fragmentation hampered swift responses during the COVID-19 crisis (48).

Global health issues are usually intertwined with economic, legal, and environmental concerns, significantly impacting global health outcomes. For instance, international trade and commerce regulations shape the distribution of pharmaceutical products (49), intellectual property laws affect access to essential medicines (50, 51), and climate change introduces new pathways for the spread of zoonotic diseases (52). Building a practical HSR framework necessitates a coordinated approach integrating health governance with these interconnected domains.

## 4 Discussion and actionable recommendations

Over the past decade, the HSR has experienced considerable challenges stemming from the vast diversity in economic development, public health governance, and medical infrastructure among its participating countries. These disparities lead to inconsistencies in healthcare needs and cooperation priorities, which show the urgent need for an extensive framework of rules and legal mechanisms to enable effective and coordinated responses. Without such a framework, responses to large-scale medical emergencies risk becoming fragmented and ineffective (53).

In response to these challenges, China has increasingly used health diplomacy to build trust and further cooperation within the HSR framework. By organizing multilateral health forums, offering training programs for partner countries’ health professionals, and facilitating regular policy dialogs, China aims to strengthen relationships and align priorities among member states. These efforts, together with joint projects tailored to local needs and public campaigns promoting “health for all,” are intended to demonstrate China’s commitment to inclusive and mutually beneficial collaboration, helping to reduce skepticism and enhance the legitimacy of the HSR as a platform for global health governance.

### 4.1 Lessons from global health governance limitations

The shortcomings of existing international institutions and legal mechanisms provide valuable guidance for the HSR’s development of a robust legal framework. Even the European Union (the most integrated regional entity with high levels of membership cohesion and shared governance) experienced significant coordination failures during COVID-19 pandemic. In the early stages of the global crisis, EU member states implemented unilateral border closures, competed for medical supplies, and withheld aid from severely affected countries (54, 55). These experiences stress the necessity for a rule-based HSR framework that includes clear protocols for resource allocation, vaccine distribution, public health facility construction, and information sharing during crises.

The most relevant legal foundation in global health is the International Health Regulations (IHR), a binding instrument authorizing the WHO to monitor and address global health risks. However, the IHR primarily focuses on detecting, reporting, and managing public health emergencies, with limited provisions for broader cooperation on health services, information and resource sharing, and long-term capacity building (56). Furthermore, the IHR has been criticized for its ambiguity in some key areas, including its notification system, criteria for declaring a Public Health Emergency of International Concern, and the absence of accountability mechanisms for participating countries that violate its rules (57).

Notably, one of the IHR’s principles shortcomings rests in its dispute settlement process, outlined in Article 56. While this article allows disputes to be resolved through negotiation, mediation, or arbitration under the Director-General’s guidance, arbitration is non-compulsory. A country may declare its acceptance of compulsory arbitration, but the IHR’s dispute settlement process is primarily non-compulsory, making it less binding on the contracting parties. This non-binding nature allows for the possibility of political interference when disputes arise. As a result, health disputes under the HSR framework would be challenging to resolve through an IHR-style framework. Uncertainty, suspicion, and hesitation may hinder international health cooperation without a rigorous and binding dispute-resolution mechanism under the HSR.

### 4.2 Compliance challenges in global health governance

Theoretically, the IHR has binding force over most HSR participating countries. However, adherence to its public health obligations has been persistently inadequate, wherein “only about one-third of the countries in the world currently have the ability to assess, detect, and respond to public health emergencies,” all elements that are expected by the IHR (58).

The WHO frequently relies on “soft law” instruments—including resolutions, recommendations, guidelines, and standards— to address global health challenges. While these tools offer helpful guidance, their lack of legal enforceability often limits their effectiveness in compelling action from non-compliant states (57). Furthermore, the authority of the WHO’s recommendations has occasionally been questioned, undermining trust and compliance. For instance, during the COVID-19 pandemic, some countries ignored the WHO’s interim guidance that “there is no reason to take unnecessary measures to interfere with international travel and trade” (59). Instead, they imposed strict unilateral measures, disrupting the flow of medical aid, personnel, and technical assistance. A lack of compliance with WHO recommendations and obligations prompted a 2021 WHO review panel to observe that non-compliance with IHR obligations “contributed to the COVID-19 pandemic becoming a protracted global health emergency” (60). These vulnerabilities should be carefully considered by China and other HSR partners when designing a governance framework.

Beyond the IHR, other international legal instruments relevant to the HSR include fundamental human rights treaties, such as the Universal Declaration of Human Rights (UDHR) and the International Covenant on Civil and Political Rights (ICCPR), as well as trade agreements like those administered by the WTO. For instance, Article XX(b) of the General Agreement on Tariffs and Trade (GATT) allows member states to adopt measures necessary to protect human health (61). The Agreement on the Application of Sanitary and Phytosanitary Measures (SPS Agreement) also promotes technical assistance for developing countries. Article 9 of the SPS Agreement states, “Each Member agrees to facilitate the provision of technical assistance to other Members, particularly developing countries, either bilaterally or through appropriate international organizations” (62). However, the SPS Agreement is limited in scope, focusing mainly on enabling countries to meet export requirements rather than broader health system strengthening.

Regarding dispute settlement, the WTO has a well-developed mechanism that has previously helped resolve health-related disputes. However, these cases generally pertain to trade rather than the complex realities of healthcare delivery. The WTO dispute settlement mechanism may be inappropriate for some health-related disputes because those disputes involve intricate interactions among governments, private entities, and consumers, and may be influenced by cultural preferences and national regulatory standards (63). As such, the WTO framework may be inefficient for resolving many health-specific disputes. The HSR can therefore learn from these limitations to construct a more effective and context-appropriate legal framework.

### 4.3 Addressing governance and regulatory flaws in the HSR framework

Theoretically, the IHR has binding force over most HSR participating countries. However, adherence to its public health obligations has been persistently inadequate, wherein “only about one-third of the countries in the world currently have the ability to assess, detect, and respond to public health emergencies,” all elements that are expected by the IHR (58).

Despite a foundation of bilateral and regional agreements, the HSR’s regulatory structure in the health sector remains fragmented and relatively limited. Most operational rules are found in memorandums of understanding (MOUs), declarations, joint statements, and implementation plans. Examples include the “Nanning Declaration on China-ASEAN Health Cooperation and Development,” the “Memorandum of Understanding between the Ministry of Health of China and the Secretariat of the League of Arab States on the Establishment of China-Arab Cooperation Mechanism in the Field of Health,” and the “Agreement on Health Cooperation between the Ministry of Health of China and the Ministry of Health of Turkey.” These instruments usually do not address the resolution of disputes arising from interpreting or implementing HSR projects. Even when they do, they usually request that states resolve disputes through consultation and negotiation within diplomatic channels without providing for legal resolution or designating legal responsibility. In short, existing legal norms and dispute mechanisms within the HSR are insufficient for rule-based governance, especially when dealing with complicated, multi-country public health crises. Enhancing existing provisions in soft law instruments, as indicated by the lessons of the COVID-19 crisis (64), is an important starting point for developing a more cohesive framework for the HSR.

#### 4.3.1 Optimizing dispute resolution, compliance, and accountability

The HSR should first of all develop an extensive dispute settlement system so as to effectively manage those already emerged disagreements, guaranteeing decisions are binding, professional and without improper political intrusion. This system could take inspiration from prevailing global dispute resolution frameworks, such as the WTO’s dispute settlement system, which emphasizes transparency and neutrality.

Additionally, HSR countries should strengthen rapid response capacities for domestic, regional, and international public health threats. This includes developing contingency plans that adapt to crises as they unfold and executing preventative and emergency operations within strict timelines. To improve compliance with preparedness and response measures, the establishment of a “compliance and accountability committee” is recommended. Such a governance body would monitor, assess, and provide detailed evaluations of members compliance. While its findings may not carry legally binding force per se, the normative pressure generated could enhance transparency and promote adherence to shared commitments (65).

#### 4.3.2 Harmonizing medical and pharmaceutical standards

A critical element of effective HSR governance is the harmonization of medical and pharmaceutical standards. Aligning these standards will streamline the circulation of medical products and advance the mobility and flexibility of qualified healthcare professionals across borders. Standardizing pharmaceutical regulations and medical accreditation processes among HSR-participating countries is essential to remove unnecessary obstacles to medical aid. These standards should align with international standards and be integrated with domestic rules and policies. Furthermore, expanding the global reach of Chinese medicine and pharmaceuticals requires domestic legal reforms to establish clear criteria for Chinese pharmaceutical enterprises to access overseas markets. This “going out” strategy should also include regulations for quality assurance, alignment with global legal standards, and the promotion of research and development centered on the actual health needs of HSR partner states.

#### 4.3.3 Developing public health norms linked to environmental health

Incorporating environmental health considerations into the HSR is essential for building long-term public health resilience. New and more specific norms should be instituted to advance public health infrastructure development and guarantee basic health protections for all HSR partner states. These norms should address the growing intersection between the right to health and environmental issues, such as pollution and climate change. Pollutant emissions cause ecological damage and climate change, leading to atmospheric warming (66). This environmental degradation exacerbates zoonotic disease risks, accelerates the spread of infectious diseases (67, 68), and undermines states’ ability to respond effectively to outbreaks amidst extreme weather conditions (69, 70). Air pollution contributes to a range of adverse health conditions, including respiratory illnesses and cardiovascular diseases (71, 72), which heighten vulnerability to respiratory viruses such as coronaviruses (MERS Cov, SARS, and common cold viruses), influenza viruses, respiratory syncytial virus (RSV), among others (73). Pollution-induced inflammation further weakens immune response and increases the risk of viral complications (74).

The COVID-19 pandemic also produced a surge in medical waste (75). China alone produced thousands of tons of extra medical waste daily, a substantial rise from pre-pandemic levels. For instance, hospitals and isolation centers in Shanghai generated approximately 1,400 tons of medical waste per day in 2022, compared to 308 tons before the outbreak (76). In this regard, the HSR should prioritize the production and distribution of recyclable and biodegradable medical products to mitigate these impacts. Investments in sustainable technologies and systems are valuable for decreasing environmental damage while advancing public health and resilient development. Clean energy, environmental protections, and green development should form the core components of HSR’s foreign health-related projects and cooperation.

#### 4.3.4 Incorporating non-state actors into governance

Non-state actors, including private sector companies and NGOs, are vital to the sustainable success of HSR initiatives. Private sector partnerships could contribute technological innovation, investment, and expertise, supporting the development of health infrastructure and efficient supply chains across HSR participating countries. NGOs could enhance HSR’s projects by engaging local communities, advocating for equitable access, and monitoring implementation to ensure transparency and accountability. The involvement of non-state actors are therefore likely to enhance the overall legitimacy, inclusiveness, and effectiveness of the HSR as they serve as observers, offer input during governmental consultations and negotiations, provide medical material assistance, and promote medical technology research and development (77). By bridging gaps between government strategies and local needs, they could particularly help tailor HSR’s projects to diverse contexts and promote more sustainable outcomes. This approach also corresponds with wider tendencies in treaty-making, which increasingly involve multi-stakeholder input (78).

Whereas, in the past decade China’s foreign health cooperation under the HSR was primarily government-to-government, with limited participation by non-State actors. During the COVID-19 pandemic, many organizations from Japan, South Korea, Cambodia, and Pakistan assisted China with epidemic prevention resources (79, 80). Though, China still lacks corresponding legal provisions to facilitate the participation of non-governmental bodies in the HSR’s foreign health assistance activities. The legal and governance framework must acclimate to promote NGO and private entity contributions by advancing clear rules to ensure their productive incorporation into foreign health initiatives.

#### 4.3.5 Integrating platforms for rule-making and financial support

Effective international rule-making relies on open communication and consultation among participating countries (81). The WHO, with its operational expertise, relatively neutral global stance, and strong reputation in health promotion, is an ideal partner for facilitating negotiations and developing international regulations on health and medicine. Closer collaboration with the WHO and comparable organizations would enhance the acceptability and effectiveness of the HSR’s governance system.

Existing multilateral and Chinese financial institutions, such as the Asian Infrastructure Investment Bank, China Development Bank, and the Export–Import Bank of China, should continue to play a significant part in financing public health systems, medical infrastructure, and pharmaceutical innovation in HSR-participating countries. These institutions can offer long-term financial support through loans, grants, and other monetary contributions. Additionally, regional platforms, such as the China-ASEAN Health Cooperation Forum, the China-Arab Countries Health Cooperation Forum, and the China-Central and Eastern European Countries Health Ministers Forum, can help integrate regional standards with international best practices, contributing to a more functional and cohesive HSR legal framework.

## 5 Conclusion

Ten years since its inception, the HSR has evolved into a significant instrument of China’s health diplomacy and soft power. Not only does it serve as a strategic extension of China’s broader foreign policy and global health ambitions, but it has also become an important platform for promoting international health cooperation and advancing the universal right to health. Though this ambitious initiative positions China as a proactive participant in addressing global health challenges while simultaneously advancing its national interests and soft power, it has substantially contributed to the development of medical infrastructure, improved access to essential medicines, effective pandemic responses, and strengthened cross-border health partnerships (82).

Nevertheless, the HSR continues to face considerable challenges that hinder its effectiveness and long-term sustainability. Significant disparities in healthcare capacity persist among HSR partner countries, and many low- and lower-middle-income nations still lack the resources necessary to safeguard public health (83). The COVID-19 pandemic further exposed difficulties in harmonizing public health strategies, fragmented policy communication, and divergent medical and regulatory standards within the HSR framework. Concerns around project transparency, financial sustainability, and potential dependency show the need for more robust risk management and stakeholder engagement mechanisms. Addressing these challenges requires the development of a more resilient, transparent, and rule-based governance framework, incorporating binding agreements on resource allocation, emergency response, and long-term health system stewardship, while aligning with international best practices and sustainability standards.

Looking ahead, the trajectory and global impact of the HSR will increasingly depend on its ability to integrate with broader multilateral health governance. To realize its full potential, the HSR should move beyond traditional bilateral approaches and embrace a “multilateral plus regional” model. This includes deepening collaboration with organizations such as the WHO, aligning with international conventions, and encouraging the participation of non-state actors—including NGOs, private enterprises, and regional platforms—to enhance legitimacy, accountability and effectiveness. By prioritizing sustainability, transparency, and inclusive governance, the HSR can serve as a global exemplar for building adaptable and resilient health systems, accelerate progress toward universal health coverage and SDG 3, and contribute to the emergence of a more just and sustainable global health order. Future research should prioritize rigorous impact assessments of HSR projects to systematically evaluate their effectiveness in improving global health outcomes, promoting equity, and strengthening health system resilience across diverse contexts. Given the existing limitations in publicly available data and the fragmented nature of reporting among HSR countries, future studies should also focus on addressing data gaps through improved transparency and standardized data collection. Moreover, stakeholder surveys (including perspectives from government officials, healthcare providers, non-state actors, and local communities) are vital for capturing on-the-ground experiences and identifying both benefits and challenges in HSR implementation. Such empirical research will not only inform evidence-based policy adjustments but also help ensure that the HSR’s future development aligns with the needs and expectations of its stakeholders, ultimately advancing SDGs and global health governance.
